# Thoracic Extraosseous Epidural Venous Haemangioma Arising From Nerve Root in a Nasopharyngeal Carcinoma Survivor: A Case Report

**DOI:** 10.7759/cureus.23421

**Published:** 2022-03-23

**Authors:** Kang Kai Lim, Sudhir Kumar, Fazir Mohamad, Dzulkarnain Amir

**Affiliations:** 1 Spine Surgery, Hospital Kuala Lumpur, Kuala Lumpur, MYS

**Keywords:** spinal cord tumour surgery, epidural spinal cord compression, extraosseous spinal venous haemangioma, thoracic epidural tumour, epidural haemangioma

## Abstract

A 66-year-old man presented to the outpatient clinic with back pain and progressive bilateral lower limb weakness over a period of 6 months. Magnetic resonance imaging showed a large extraosseous epidural lesion at T6-T7 arising from the left T6 spinal nerve root complicated with cord compression leading to cord oedema. The lesion was excised en bloc and histopathological examination revealed benign venous haemangioma. We report this rare case of venous epidural haemangioma to be considered as a differential diagnosis in a patient with a background of previous lumbar discectomy surgery and who was a nasopharyngeal carcinoma survivor.

## Introduction

Haemangiomas are mostly benign blood vessel tumours that can form on the skin, tissues below the skin or in an organ. Vertebral haemangiomas involve the proliferation of normal capillary and venous structures in the marrow of the vertebral body. Such lesions are commonly an incidental finding upon radiological workup. Patients are usually asymptomatic or they may have a varying degree of back pain. Intraosseous vertebral haemangiomas represent the commonest benign tumour of the spine which are usually described as neoplasm of endothelial cells that grow inside the marrow of the vertebral body as they are vascular in origin. Extraosseous spinal haemangiomas are extremely rare and an aggressive growth within the epidural space can cause compression of neural elements and result in neurological deficit. Unlike intraosseous haemangiomas, which can be detected by plain radiographs or CT scans, extraosseous haemangiomas are more elusive and are usually only evident on MRI scans. It is vital for the treating physician to consider the differential diagnosis of extraosseous epidural haemangioma (EEH) when encountering patients with apparently normal radiographs or CT scans, especially in patients with multiple distracting red herrings.

## Case presentation

We report a case of a premorbidly active 66-year-old male who complained of progressive lower limb weakness associated with numbness below the umbilicus for six months. The bilateral lower limb numbness was accompanied by mid-thoracic back pain which was preceded by a trivial fall from the ladder 3 months prior. His symptoms progressed with difficulty in micturition requiring straining. He was also diagnosed with nasopharyngeal carcinoma 10 years ago and was a survivor having completed treatment with no relapse thus far. He had an open lumbar discectomy done five years prior to presentation in another centre for radiculopathy. He denied significant loss of weight and has a normal appetite. There was no prolonged fever. He was able to stand and ambulate short distance with a walking frame at the time of consult with Medical Research Council (MRC) grade 3/5 for right lower limb and grade 4/5 for left lower limb. Bilateral knee and ankle reflexes were hyper-reflexic but there was no sustained ankle clonus. His abdominal reflex was absent. He had sensory impairment below T9 level. His tumour and infective markers were not remarkable. His other blood panels were within normal range.

**Figure 1:**
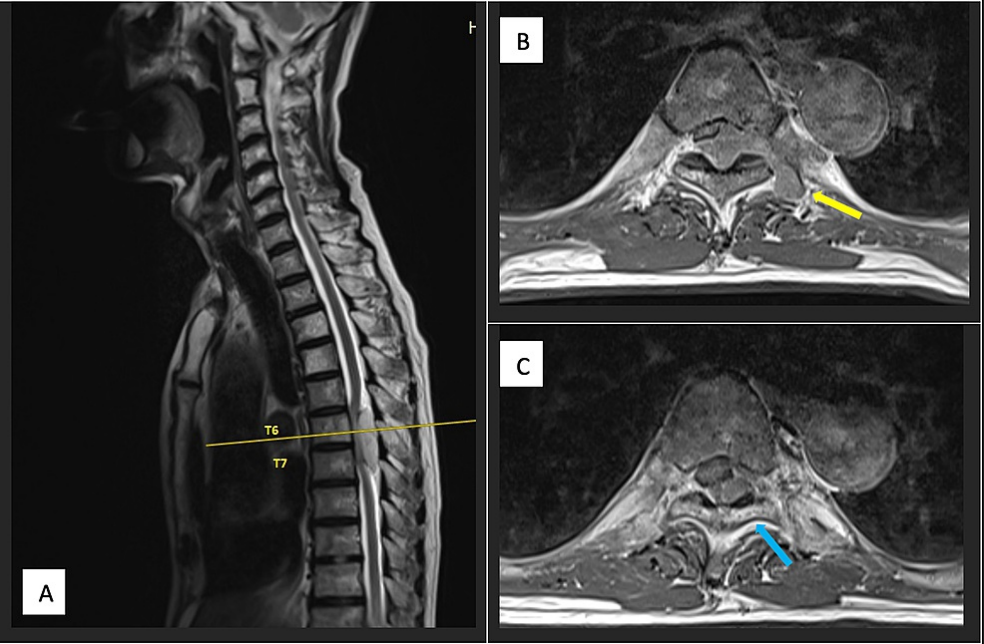
(A) Sagittal T2-weighted MRI showing a soft tissue tumour spanning the T6 to T7 epidural space. (B) Axial MRI image at T6 showing tumour exerting mass effect on the spinal cord with left neuroforamen extension forming a “dumbbell-like” tumour (yellow arrow). (C) Axial cut at T7 showing tumour extension (blue arrow).

An MR scan (Figure 1) was ordered and he was found to have a large extraosseous epidural soft tissue lesion spanning the level of T6 to T7 (Figure 1A). The spinal canal was narrowed to 0.4 cm and the thoracic spinal cord was compressed leading to cord oedema (Figures 1A-1C). The left T6 spinal nerve root was also encased by this mass in a dumbbell-like fashion (Figure 1B).

**Figure 2:**
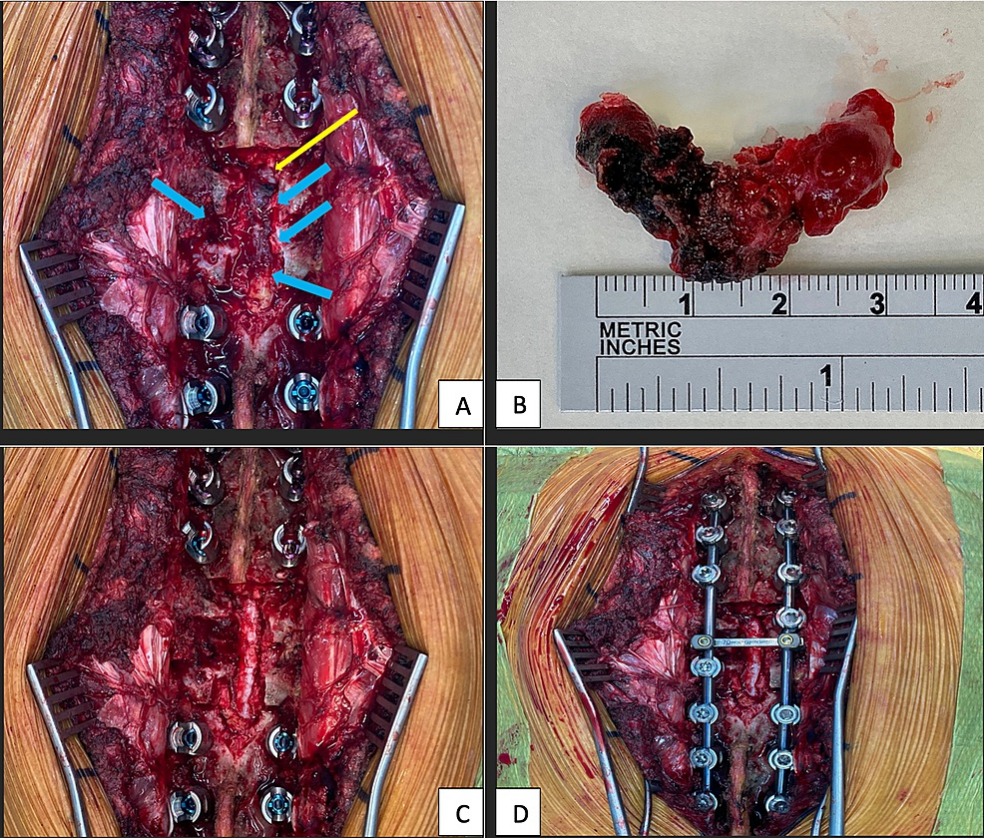
(A) Epidural tumour (thick blue arrows) seen encroaching the dura (thin yellow arrow) and extended into the left neural foramen post-laminectomy. (B) Tumour excised en bloc measuring 3 cm × 2.2 cm. (C) Tumour removed and the dura is free. (D) Final construct of instrumentation.

**Figure 3:**
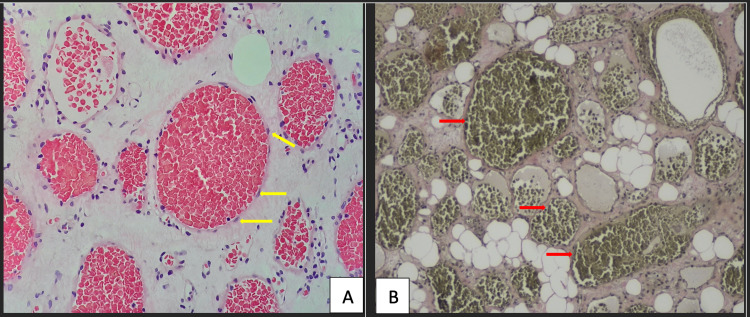
(A) Haematoxylin and eosin (H&E) stained photomicrograph at ×40 objective magnification showed numerous vessels lined by benign endothelial cells (yellow arrows). (B) Elastin van Gieson (EVG) stain showed vessels from the excised tumour lacking elastic fibres within the walls (red arrows), confirming the diagnosis of venous haemangioma.

The patient underwent decompressive laminectomy and resection of the tumour and instrumented spinal stabilisation. Intraoperatively, a soft tissue mass of 30 mm × 22 mm × 15 mm was seen compressing on the dura and encroaching into the left T6 neuroforamen following laminectomy (Figure 2A). The soft tissue tumour was removed en bloc and sent for a histopathological exam (HPE) to exclude spinal metastasis (Figure 2B). Haemostasis was secured with bipolar sealer (Aquamantys TM, Medtronic, Dublin, Ireland). HPE of the excised tumour revealed tissues composed of numerous vascular channels with absent elastic laminae, consistent with venous haemangioma (Figures 3A-3B). There was no nuclear atypia or evidence of malignancy seen. A diagnosis of T6 extraosseous epidural venous haemangioma was made.

**Figure 4:**
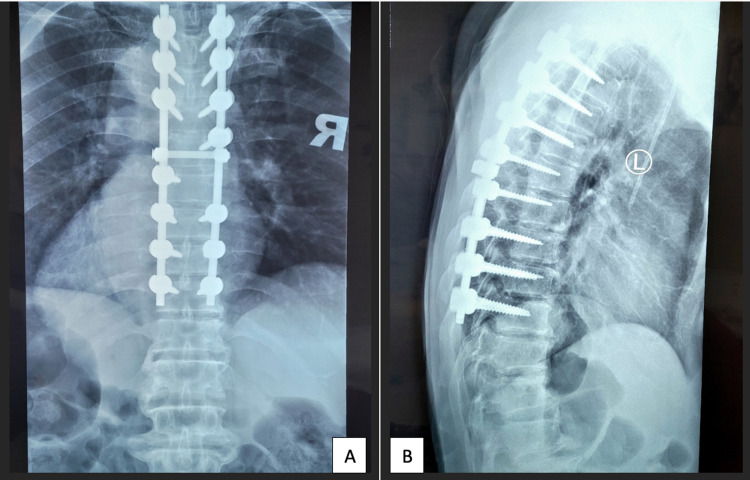
Postoperative 1-month check radiographs: (A) Anteroposterior view. (B) Lateral view.

A check radiograph at 1-month postoperative revealed stable instrumentations (Figures 4A-4B). Our patient regained his motor functions uneventfully and was able to walk unaided at 3-month postoperative.

## Discussion

Vertebral osseous haemangiomas with extraosseous extension are rare entities but they are widely reported in comparison to solitary EEHs [1-3]. EEHs are extremely rare and usually present as incidental findings on radiological diagnostic imaging such as MRI and CT scans. Acute presentation is rare and as seen in our case, patients with EEH complain of progressive myelopathy or gait disturbance for a prolonged period of time. Other presenting symptoms of these patients include on and off acute back pain that does not resolve with regular analgesics and radiculopathy due to compression.

Extraosseous epidural venous haemangiomas are often described by their radiological findings as a cyst-like mass in comparison to cavernous or capillary haemangiomas as a solid hypervascular mass [4]. This is further proven with a low-intensity signal on T1 and hyperintense on T2 of the MRI, which is vital to assess radiologically if there is any concomitant cord or nerve root compression. However, we found this is not always the case as our patient’s MRI showed a solid epidural soft tissue mass. The other differential diagnoses for such lesions from radiological findings are lymphomas, meningiomas, dural fistulae, pure epidural haematoma, and in some cases, herniated disks [4]. This is later on confirmed via histopathology examination if excision of the lesion is done.

This type of haemangioma due to its progressive presentation of symptoms will require some form of management, may it be via surgery, interventional radiology for embolisation, oncological treatment or a combination of treatment as there is no consensus at the current time for its management. However, due to its nature of being extraosseous and highly vascular, a biopsy can prove tricky and difficult. Any attempt to attain a soft tissue biopsy may risk an accumulation of haematoma which may worsen neurological status. Laminectomy and a complete haemangioma excision followed by spinal instrumentation for stabilisation have proven to be successful in our case. Given their inherent nature, haemangiomas pose a high intraoperative risk of uncontrolled bleeding due to their dense vascular structures and thus surgeons should pre-empt this by making sure an armamentarium of haemostatic products and techniques are available at hand. The risk of dural involvement such as adhesion and tear should also be considered, and appropriate intraoperative monitoring should always be on board. Controversies surrounding preoperative embolisation is due to the involvement of the watershed area in the thoracic spine which may potentially be one of the main blood supply vessels to the spinal cord. In some cases, due to its highly radiosensitive nature, treatment of the pain using radiotherapy has proven useful and, in some cases, has shown good outcomes in resolving the paralysis which it causes in patients. Some literature showed that it is used as first-line treatment in aggressive and recurrent haemangiomas [3]. Postoperative radiotherapy has also shown an overall improvement in symptoms, however, long-term studies are lacking in showing that this option of treatment can be used safely as a gold standard.

## Conclusions

Although intraosseous vertebral haemangiomas are the commonest benign tumours of the spine, in rare cases, an extraosseous extension of these aggressive haemangiomas into the epidural space can cause neurological events. In contrast, EEHs can mimic malignant metastasis in the spine without any bony involvement. A neurological deficit can be marked permanent if operative removal is delayed. Tissue biopsy of these rare haemangiomas can prove difficult, and often, surgical intervention is usually needed. A differential diagnosis of EEH can help surgeons better prepare the patient for surgical intervention.
